# Resistance of *Streptococcus Pneumoniae* to Macrolides in Iran

**Published:** 2019-02

**Authors:** Shervin Shokouhi, Ilad Alavi Darazam, Atousa Yazdanpanah

**Affiliations:** 1 Infectious Diseases and Tropical Medicine Research Center, Shahid Beheshti University of Medical Sciences, Tehran, Iran.; 2 Department of Infectious Diseases and Tropical Medicine, Loghman Hakim Hospital, Shahid Beheshti University of Medical Sciences, Tehran, Iran.

**Keywords:** Antimicrobial/resistance, Streptococcus pneumoniae, macrolides, Iran

## Abstract

**Background:**

Antimicrobial resistance of *Streptococcus pneumoniae* (*S. pneumoniae*) has shown major changes in recent years. On the other hand, macrolide antibiotics are being increasingly used in clinical practice. Several studies have reported increased resistance to this group of antibiotics, while there is no comprehensive information in this area. Accordingly, the present study was designed to estimate the resistance of *S. pneumoniae* to macrolides in Iran.

**Materials and Methods:**

In this review, articles (2000–2017), evaluating the level and type of *S. pneumoniae* resistance to macrolides in Iran, were extracted by searching different databases, and the results were analyzed.

**Results:**

A total of 25 relevant articles were retrieved and analyzed. Overall, 2723 cases had been recruited in these studies. The mean percentage of resistance to macrolides was estimated at 48.43% (CI, 38.8–57.9%). In the majority of reported cases, the resistance mechanisms included ribosomal methylation (i.e., ermB mutation), dual resistance, and efflux-mediated resistance.

**Conclusion:**

Based on the findings, the resistance rates are considerable in different cities of Iran. Therefore, without determining the type of drug resistance in clinical samples, use of macrolides is not recommended for treatment purposes. In addition, considering the type of resistance mechanisms in Iran, use of higher drug doses is probably ineffective.

## INTRODUCTION

*Streptococcus pneumoniae* (*S. pneumoniae*) is one of the main causes of Community-Acquired Pneumonia (CAP), meningitis, sinusitis, otitis media, and sepsis (1). The commensal *S. pneumoniae* pathogens are known to cause nasopharyngeal colonization (1). The introduction of penicillin in the 1940’s revolutionized the treatment of diseases caused by this micro-organism, and for many years, many bacterial infections were easily treated with this agent.

Penicillin-resistant cases and treatment challenges started to emerge in the 1980’s and 1990’s (1, 2). Today, despite the emergence of new classes of antimicrobial drugs, *S. pneumoniae* remains one of the most important causes of mortality, especially in children (3). The prevalence of penicillin-non susceptible *S. pneumoniae* varies in different regions, ranging from 0–5% in England, Germany, and some Scandinavian countries to 25–50% in Spain, France, and Greece before 2009 (4).

Macrolides are a class of antibiotics, which have been used for many years as a treatment for pneumococcal infections. These drugs reversibly bind to 50S ribosomal subunit and inhibit RNA-dependent synthesis of proteins. The widespread use of this class of drugs has led to a gradual increase in resistance rates. The resistance mechanisms against these agents are diverse and include ribosomal target site alteration, alteration in antibiotic transport, and antibiotic modifications (1). Each of these mechanisms has a different genetic basis.

Two main mechanisms, i.e., efflux-mediated drug resistance and ribosomal target site alteration, are encoded by *mef(A)* and *erm(B)* genes, respectively; resistance may also occur due to a combination of these mechanisms (4). It seems that each of these mechanisms causes a different level of resistance. Changes in the *erm* gene, which modify 23S ribosomal RNA, cause high levels of resistance, while the efflux-mediated mechanism leads to medium to low levels of resistance (> 64 g/mlM1C). Overall, according to previous studies, the relative distribution of these 2 mechanisms varies greatly in different parts of the world.

Since the selection of treatment strategies depends on the involved resistance mechanism, further information seems necessary for determining the type of resistance in clinical practice. The highest prevalence of macrolide resistance has been reported in the Far East (Taiwan, 98%; South Korea, 88%; Japan, 78%). Based on previous studies, the prevalence rates vary among countries in the same continent or even neighboring countries. For instance, in Greece and Turkey, as neighboring countries, the prevalence rates have been estimated at 29 and 2%, respectively (5). In addition, according to different studies, the prevalence of resistance varies greatly in Western Europe, ranging from 48–58% in France to 17% in Germany and 9% in England (5).

It seems that resistance to macrolides is associated with the use of these antibiotics in each region. For instance, low resistance rates have been reported in Northern European countries, whereas the prevalence of resistance is quite high in Southern and Eastern Europe (6). In addition to major differences in macrolide resistance in different countries, multiple types of resistance have been identified, as well. In the United States, a comprehensive review in 2000–2003 found mef-mediated resistance to be the major resistance type.

It seems that resistance types are changing, considering the increased number of cases with both erm(B) and mef(A) mutations despite the decreased prevalence of mef(A) resistance (7). However, erm(B)-mediated resistance is recognized as the predominant mechanism throughout the world (1). In this regard, a meta-analysis performed in 2015 on the prevalence of pneumococcal nasal carriers reviewed 6 studies related to erythromycin resistance and reported a resistance rate of 30% (CI, 10–49%) (8).

Overall, the results of previous studies on estimation of antimicrobial resistance are helpful but inadequate, as many findings have been unexpectedly rejected due to different factors. Another problem is that the collected samples and resistance mechanisms have not been fully examined in some previous studies. Therefore, this review was performed considering the importance of *S. pneumoniaee* as one of the main causes of CAP and the major role of resistance to macrolides (as one of the most common antibiotics) in the process of clinical decision-making.

## MATERIALS AND METHODS

Literature review was carried out in databases, including Google Scholar, Web of Sciences, Scopus, PubMed, Magiran, and SID. Keywords including “*Streptococcus pneumoniae*”, “pneumococcus”, “macrolides”, “Iran”, “Asia”, “antimicrobial resistance”, “bacterial resistance”, “microbial resistance”, and “drug resistance” were searched either separately or in combination.

All articles written in English or Farsi (published since 2000) were retrieved. Studies, which did not address *S. pneumoniae* resistance to macrolides, were excluded from the analysis. On the other hand, articles with other objectives, which provided information on macrolide resistance, were examined. Studies including less than 10 cases were excluded from the analysis. In addition to articles retrieved with this method, the reference lists were reviewed, as well. Overall, studies which contained relevant and unrepetitive information were included in the final analysis.

The required information, including microbial resistance pattern, method of resistance identification, study population, duration, type of clinical specimens, type of study sample (patients or healthy people), and location of the study, was extracted and studied independently.

## RESULTS

According to the research method, 25 articles were considered relevant and eligible after detailed examination. In total, 2723 cases had been analyzed in these studies (minimum, 12; maximum, 573). The mean number of cases in the retrieved articles was 116.7±108.9. In total, 8 studies included children, 2 were performed on adults, and 4 were carried out on both age groups (unidentified in 5 articles).

The majority of studies (14 articles) were conducted in Tehran, followed by Zahedan (3 articles), Shiraz (2 articles), Birjand, Hamadan, Kashan, Kermanshah, Shahrekord, and Yazd. The results are presented in Table 1.

**Table 1.** Summary of studies

	**City**	**Population**	**Case number**	**Period of time**	**Macrolide resistance**	**Other resistance charactristics**	**Lab method**	**Full description of samples**	**Excluding previous AB treatment**	**Reference**
1	Tehran	Children	194	2001–2011	35	Susceptibility to erythromycin declined from 75% (12 of 16) in 2001 to 35% (15 of 43) in 2011	Disk diffusion method	Blood, sputum, eye, CSF, ascites, ear and others	?	(9)
2	Hamadan	?	55	2014	21.8	21.8% were resistant to macrolide with the following genotypes: ermB+ (n=6, 10.9%), mefA+ (n=10, 18.2%), ermB+ mefA+ (n=4, 7.3%), ermB- mefA- (n=35, 63.6%). According to the E-Test, 25.5% of the isolates were resistant to erythromycin, 18.2% to clarithromycin, 16.4% to azithromycin.	E-Test PCR	CSF, sputum, otorrhea, pharynx, ear and eye	?	(10)
3	Tehran	Children and adults	73	2012–2015	83.6		Broth microdilution method	Cerebrospinal fluid, blood cultures, sputum, BAL, eye, nasal and other body sites (brain abscess, joint fluid, throat, abdominal fluid, ear)	?	(11)
4	Tehran	?	186	2011–2013	47.5	Antibiotic susceptibility results for erythromycin revealed that 43 (46%) from clinical and 45 (49%) from normal flora isolates were resistant to erythromycin. The double-disc diffusion test revealed that 74 (84%) indicated the cMLSB phenotype, and 14 (16%) were assigned to the M phenotype. Amongst the isolates, erm(B) gene alone was detected in 39 (44%) and mef(A/E) in14 (16%) isolates, while 35 (40%) isolates harbored both erm(B) and mef(A/E) simultaneously. The erythromycin MIC for 84% of isolates with cMLSB phenotype was ≥256 μg/mL. The MIC for all of isolates with the M phenotype was 1.5–16 μg/mL. MLSB phenotype (MIC >256 μg/ml) (80%) and 12 isolates M phenotype (MIC of 1.5 to 16 μg/ml) (20%)	Disk diffusion method Double-disc diffusion method (D test) PCR with two sets of primers specific for erm(B), mef(A/E), and tetM Disk diffusion method	Clinical and normal flora isolates	Yes, 6 months	(12)
5	Tehran	Children and adults	100	2011–2013	60	The ermB, ermB/mef and mef were carried in 50%, 36% and 18% of the clinical isolates, respectively. Amongst the erythromycin resistant nasopharynx normal flora, 47%, 28% and 25% were ermB, ermB/mef and mef, respectively.	Double disk diffusion method (D test) PCR amplification of ermB, mefA/E (mef)	Clinical and normal flora isolates	?	(13)
6	Tehran	Children	53	2013–2016	71.4		Broth microdilution method	Blood and cerebrospinal fluid	?	(14)
7	Tehran	?	46	1996–2000	78		Disk diffusion method	Blood	?	(15)
8	Tehran	?	162	2010–2013	46	The results of the E-test showed that minimal inhibitory concentration (MIC) was between 2 and greater than 256 Kg/mL.	E test Disc diffusion method E-test method for erythromycin	Clinical and normal flora isolates	Yes, 6 months	(16)
9	Tehran	Children and adults	88	2011	52	37 of 47 of tested isolates were cMLS, and 10 were M phenotypes. erm(A) gene was not detected at all. All cMLS isolates had the erm(B) gene, and all M phenotypes had the mef genes. The mef gene was also found in 6 erm(B)-positive isolates.	Double-diffusion disc test Multiplex PCR for erm(A), erm(B), and mef(A/E)	blood (15 samples), eye (9), sputum (8), cerebrospinal fluid (6), trachea (3), otitis media (3), bronchoalveolar lavage (1), pleural fluid (1), and sinus aspirates (1)	?	(17)
10	Tehran	2–26ys	60	2014–2015	67	-	Disk agar diffusion Disk diffusion method	Myringotomy	Yes, 2 weeks	(18)
11	Zahedan	10–19 ys	136	2007–2008	18.3	-	Micro broth dilution method	Normal flora isolates	?	(19)
12	Tehran	1–10 ys	573	2008–2009	56.05	-	Disk diffusion method	Normal flora isolates	Yes, 2 weeks	(20)
13	Shiraz	Children and adults	115	2001	18.3	-	Microbroth dilution method	cerebrospinal fluid, blood, sputum and nasal secretion from patients	?	(21)
14	Shiraz	Children	12	2007–2008	10	-	Disk diffusion method	Myringotomy	?	(22)
15	Zahedan	≤14 ys	75	2008–2010	90.7	-	Disk diffusion method Micro broth dilution method	Blood, cerebrospinal fluid, pleural fluid, other sterile material such as peritoneal pericardial fluids	?	(23)
16	Kashan	7–19ys	291	2011–2012	8.2	-	Disk diffusion method	Normal flora isolates	Yes, <15 days	(24)
17	Kermanshah	2–12 ys	83	2012	63.9	-	Disk diffusion method	Nasopharynx	Yes, 1 months	(25)
18	Tehran	Children and adults	15	2010–2011	65	-	Disk diffusion method	Acute otitis media	Yes, 2 weeks	(26)
19	Zahedan	10–19 ys	136	2000	18	-	Micro broth dilution method	Nasopharynx	?	(27)

## DISCUSSION

The results of the present study showed that resistance to macrolides is considerable in different parts of Iran. According to our literature review, half of previous studies have reported prevalence rates of more than 50%. On the other hand, similar to other countries around the world, especially European countries, 23S rRNA methylation was the dominant mechanism in the development of macrolide resistance. In addition, efflux-mediated resistance (independent or in combination with *ermB* gene) was considerable, as well. In this regard, a study by Haghi Ashtiani et al., evaluating the trend of changes in resistance over 10 years, showed that levels of antibiotic resistance are increasing rapidly. For instance, resistance to macrolides, such as erythromycin, has increased from 25 to 65% (9).

Among studies on macrolide resistance of pneumococci among patients with clinical syndromes and healthy subjects with normal flora, only 1 case was independently reported, thus revealing no significant difference between the 2 groups. In addition, there was no significant difference in the level of resistance among studies evaluating normal nasopharyngeal flora and clinical samples of patients; distributions were also found to be similar (12, 13, 16).

Overall, 17 studies had applied the disk diffusion method for determining macrolide resistance, while in 6 and 2 studies, broth microdilution and agar dilution were carried out, respectively. In addition, only 3 studies had applied Etest, and the results revealed no significant difference from other methods regarding the resistance rates (10, 16, 17). In 4 studies which had used the Polymerase Chain Reaction (PCR) method to determine the main genes involved in macrolide resistance, *erm(B)* gene was the only predominant gene, accounting for 10.9 to 78% of cases.

In contrast, *erm(A)* gene has not been reported in any previous study (10, 12, 13, 17). According to the literature, *mef(A)* gene has been found in 16–25% of infected cases, while combination of *mef(A)* and *erm(B)* genes has been reported in 7.3–40% of evaluated cases. These findings are associated with increased penicillin resistance, probably due to the extensive use of antibiotics in the country. In addition, emergence of new types of macrolides, such as clarithromycin and azithromycin, over the past decades, has led to their extensive use for a wide range of clinical problems, especially upper respiratory tract infections without any bacterial cause.

Previous studies have also shown that macrolide resistance is completely dependent on the geographical region, with rates ranging from nearly 10 to 100% (1). This variability is obvious in studies performed in different cities of Iran. However, the results generally confirm the high level of resistance to macrolides (9–33). Geographical location can also partly influence the type of mutation and resistance.

Except erm(A) and erm(TR), which are rarely found in pneumococcus isolates, erm(B) mutation is predominant in most mutational domains, as already stated, and is directly associated with high levels of macrolide resistance; for instance, erythromycin Minimum Inhibitory Concentration (MIC) usually exceeds 256 μg/mL (1).

In studies performed in Iran on the type of mutations, methylation-related mutations almost followed the same pattern; erm(A) was not observed in any of the samples, while erm(B) was the main mutation (10, 12, 13, 17). Efflux-mediated mechanism of macrolide resistance has been also documented in several studies. In fact, it is the dominant mechanism in Northern American countries, England, Ireland, and Argentina (1).

On the other hand, dual resistance (simultaneous presence of *erm* and *mef* genes) seems to have increased over the past years. However, the rates of resistance vary greatly in different countries. In some regions, such as Turkey (44.4%), Greece (32%), South Korea (43.3%), China (30–37%), and South Africa (46.4%), high prevalence rates have been reported over the past decade, whereas in countries, such as Thailand, Malaysia, Japan, Austria, Denmark, Saudi Arabia, Ireland, Germany, Poland, Italy, Switzerland, and England, the rates are quite low (<10% or even 0%) (1). Based on the literature review in the present study, the prevalence of resistance is relatively high in Iran, except for one study reporting a prevalence rate of < 10% (10, 12, 13, 17).

## CONCLUSION

In general, macrolides are not suitable options for clinical use against *S. pneumoniae*, given their widespread use in Iran (available to patients without prescription) and high resistance of pneumococci to these antibiotics. Therefore, use of macrolides in clinical settings, without knowledge of resistance patterns, can be risky. In addition, considering the type of resistance to macrolides (dominance of methylation mechanism or dual resistance), use of higher drug doses is not a suitable method for preventing efflux-mediated resistance in Iran. In addition, monitoring of antibiotic use and prescription should be reviewed and revised, and more serious and practical measures need to be adopted.
